# Pushing Optical Resolution to the Few-Nanometer Scale via dSTORM Imaging of Expanded Specimen–Gel Composites

**DOI:** 10.3390/gels11070491

**Published:** 2025-06-25

**Authors:** Jimmy Ching-Cheng Hsu, T. Tony Yang

**Affiliations:** 1 Department of Electrical Engineering, National Taiwan University, Taipei 10617, Taiwan; r12945027@ntu.edu.tw; 2 Graduate Institute of Biomedical Electronics and Bioinformatics, National Taiwan University, Taipei 10617, Taiwan

**Keywords:** superresolution microscopy, SMLM, dSTORM, expansion microscopy (ExM)

## Abstract

Direct stochastic optical reconstruction microscopy (dSTORM) circumvents the diffraction limit of light, emerging as a powerful superresolution technique for visualizing subcellular structures with a nanoscale resolution of 10–20 nm. Yet achieving ultrastructural resolution using dSTORM alone remains challenging, despite its advantage of requiring only minimal modifications to the imaging setup and sample preparation compared to conventional fluorescence microscopy. A recent advancement that integrates expansion microscopy (ExM), which embeds specimens in a swellable polymer gel, with dSTORM holds promise for attaining imaging resolutions below 10 nm. The combined resolution, however, is governed by the expansion factor of samples, and prior studies have primarily focused on integrations involving approximately 4-fold gel expansion, as dSTORM imaging of high-fold-expanded specimens is still technically demanding. Here, we propose a pragmatic expansion strategy—post-labeling ten-fold robust expansion microscopy (plTREx)—and outline a workflow to facilitate its compatibility with dSTORM, collectively termed plTREx-dSTORM. Specifically, this workflow enhances the mechanical stability of the expansion hydrogel and improves fluorescence signal density across both widefield and dSTORM imaging platforms. Furthermore, we optimize the re-embedding protocol to integrate hydrogel expansion with dSTORM while preventing gel shrinkage. Together, plTREx-dSTORM enables highly refined imaging capable of ultrastructural interpretation of cellular proteins, effectively bridging the resolution gap between electron microscopy and optical microscopy.

## 1. Introduction

Light microscopes are essential for biological studies at different scales—tissues, cellular, and molecular levels. Fluorescence microscopy is a powerful imaging technique that enables the visualization of protein localization, dynamic biological processes, cell-to-cell communication, and subcellular structures. However, due to the wave nature of light, structural features smaller than ~200 nm cannot be resolved using conventional optical systems, as described by both Abbe’s and Rayleigh’s criteria for optical resolution in a diffraction-limited system [1,2].

The advent of superresolution microscopy has revolutionized optical imaging for nanometer-scale samples by circumventing the diffraction limit, enabling the imaging of samples with resolution not achievable via traditional optical microscopy, such as confocal or widefield microscopes, and achieving target specificity with labeling strategies not easily found in electron microscopes [3,4,5,6,7,8,9,10,11,12,13]. Superresolution imaging provides insights into biological structures and small molecules at the nanometer scale. Specifically, this imaging technique is an umbrella term that encompasses three main categories: structured illumination microscopy (SIM) [9,11,12,14,15], stimulated emission-depletion microscopy (STED) [4,10,13], and single-molecule localization microscopy (SMLM) [3,5,7,8,16,17,18]. These methods, in addition to their resolving power below 200 nm, keep the same advantages as light microscopy in specimen preparation, specific labeling via dye-conjugates, and the degree of freedom in multicolor imaging.

Among the superresolution techniques, SMLM, a collection of techniques, has the exceptional ability to visualize specimens at a higher spatial resolution than other superresolution methods, principally working by only allowing a few fluorophores in the fluorescent state (“ON” state) while keeping the other in the non-fluorescent state (“OFF” state) [3,5,7,8,16,17,18]. dSTORM exploits the photophysical properties of fluorophores by stochastically switching the ON (for only a fraction of fluorophores) and OFF (for most fluorophores) states, with the principle that the imaging buffer initiates the oxidation-reduction reaction for activating the on-and-off switching mechanism in the organic fluorophores [3,7,16]. With the few fluorescent molecules in each time frame, one can find a precise location of the centroid of their point-spread function (PSF) in each image. Now, by repeated imaging, accumulated fluorophore localizations can be reconstructed in the superresolution image, with a spatial resolution of approximately 10–20 nm.

However, protein molecules are typically only a few nanometers in size, and even with the resolution of dSTORM, accessing finer structural features, such as protein arrangements and relative molecular localizations, remains challenging. To overcome these limitations, researchers have sought complementary strategies to enhance effective resolution. One promising solution is to physically expand the sample within the polyacrylamide gel matrix, a method known as expansion microscopy (ExM) [19,20]. This leads to integrating ExM with superresolution techniques such as STED and SMLM, forming the hybrid approaches Ex-STED and Ex-SMLM, respectively. Specifically, due to high spatial resolution in SMLM imaging, Ex-SMLM allows us to access finer details in the structure that cannot be easily achieved using ExM or SMLM alone. Recent efforts have robustly employed four-fold expansion strategies with dSTORM and STED [21,22,23], and these strategies have significantly advanced the understanding of nanoscale protein organization, spatial relationships between biomolecules, and the structural composition of cellular organelles [19,20,24]. However, in intricately organized structures, such as centrioles, even four-fold expansion can result in overlapping localization signals and insufficient spatial separation between molecular targets.

We sought to further the effective resolution directly associated with the degree of expansion in Ex-SMLM in imaging by developing a refined Ex-SMLM method. To this end, we turned to Ten-fold Robust Expansion Microscopy (TREx) [25,26], a member of the ExM family that achieves higher expansion factors while maintaining tissue and cellular integrity. Building on this, we introduce a post-labeling variant of TREx integrated with dSTORM, which we term plTREx-dSTORM. This can theoretically enhance the ability to visualize nanometer-scale specimens with high fidelity and achieve an effective spatial resolution of a few nanometers.

However, we encountered several issues in developing plTREx-dSTORM. First, the TREx expansion sample is fragile and easily breakable, which makes it difficult to manipulate (Supplementary Figure S1a). Moreover, we found that cell integrity may be compromised (Supplementary Figure S1b). Second, the fluorescent signals of the TREx-expanded sample are too weak to display the protein architecture, resulting in a loss of fluorescent signals (Supplementary Figure S2). This is particularly problematic for marker proteins such as ATP synthase, which we previously used as in situ references for drift correction in dSTORM imaging [22,27]. Third, unoptimized re-embedding conditions may not ensure maximal retention of the expansion factor when specimen-gel composites are incubated in imaging buffer solutions.

**Figure 1:**
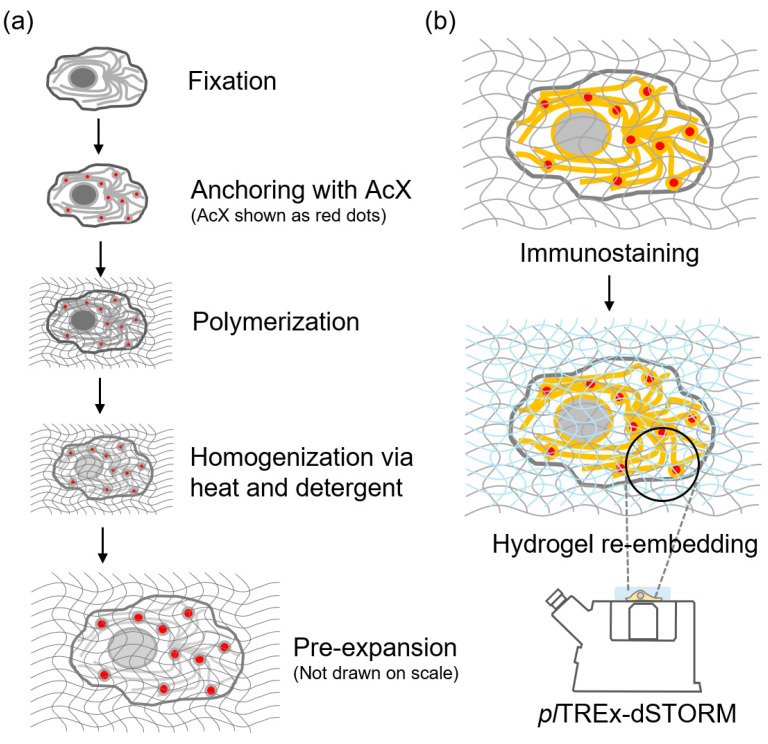
The workflow of pl-TREx-dSTORM. This figure lays out the experimental steps of preparing expansion hydrogel (**a**) and post-hydrogel modification (**b**). (**a**) shows the general hydrogel preparation of pl-TREx––fixation, anchoring via Acryloyl X CE (AcX in short), polymerization, homogenization via heat and detergent, and ten-fold expansion. (**b**) displays the hydrogel handlings after expansion. Immunostaining or other staining methods are available to specifically label biomolecules of interest. Then, hydrogel re-embedding to sustain the specimen’s structural integrity in the dSTORM imaging buffer. Note that the diagram is not drawn on scale.

Therefore, this study was pinpointed with several troubleshooting areas and developed a pragmatic method by post-labeling the sturdy expansion hydrogel to preserve structural fidelity, refining the homogenization process to improve fluorescent signal output, and achieving 10-fold expansion under harsh dSTORM imaging conditions (Figure 1). With the combinatorial imaging solution, we can visualize the miniature architecture that governs essential biological functions inside cells. Thus, this study aims to enhance superresolution imaging using the plTREx-dSTORM system by elucidating the structure of interest, specifically centriolar proteins. The plTREx-dSTORM serves as an effective approach to investigate structural and molecular networks in biological systems at the few-nanometer scale.

## 2. Results

### 2.1. Development of Post-Labeling TREx

To overcome the challenges identified in the original TREx workflow and its combination with dSTORM (Supplementary Figures S1 and S2), we focused on mechanical reinforcement of the hydrogel during polymerization, a denaturation-based homogenization method to preserve the specimen’s structural integrity more effectively, and a post-labeling strategy to retain fluorescent signals for single-molecule localization microscopy, where fluorescence is crucial for generating superresolution images.

**Figure 2:**
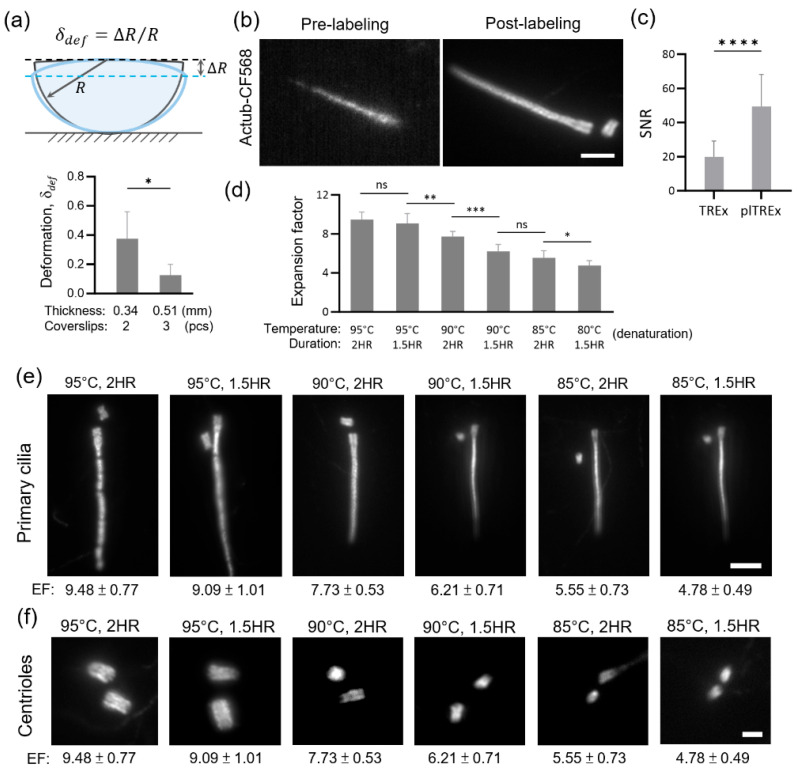
Post-labeling TREx (plTREx) performance evaluation. (**a**) Deformation analysis. Accessing the deformation of gels made by stacking two or three coverslips in the gelation environment (refer to Supplementary Figure S3) to make 0.34 mm and 0.51 mm-thick gels. Data are presented as mean ± s.d., based on 5 independent experiments each. (**b**) Structural display of primary cilium in different staining timing and condition. Primary cilium characterized with AcTub-CF568 antibody conjugate in pre-staining followed by proteinase-k digestion treatment or post-staining followed by heat-induced denaturation treatment. (**c**) Statistical analysis of signal-to-noise ratio (SNR) from pre-TREx and post-TREx data. Data are presented as mean ± s.d., based on 15 measurements. (**d**–**f**) Expansion factor analysis of centriolar proteins in different denaturation conditions. (**d**) shows the statistical data of expansion factor analysis (*N* ≥ 10 for each condition). Data are presented as mean ± s.d. (**e**,**f**) Each panel shows the structures of primary cilia and centrioles under different conditions analyzed in (**d**). Unpaired two-tailed *t*-test was used to assess statistical significance. Significance is indicated as follows: *p* < 0.05 *, *p* < 0.01 **, *p* < 0.001 ***, *p* < 0.0001 ****; ns, not significant. Scale bar, 10 µm in (**b**), 5 µm in (**e**), and 2 µm in (**f**).

To begin with, we addressed the fragility of hydrogels by redesigning the gelation environment (Supplementary Figure S3). In the previous gelation setup, TREx hydrogels were too delicate and fragile, making them difficult to manipulate and prone to tearing, which limited their transferability and imaging. This further leads to compromised cell integrity, as evidenced by the multiple discontinuities in the cytoskeleton (Supplementary Figure S1b). To mitigate these issues, we physically introduced a gelation chamber that enhances the gelation environment, with each hydrogel formed to a thickness of approximately three standard coverslips. The setup is simple yet effective in strengthening the structural rigidity of the hydrogel matrix. Following the hydrogel deformation analysis using the original TREx technique, we measured hydrogels with different thicknesses. We found that, under our setup, hydrogels exhibited a deformation rate of 0.03 compared to 0.2 in the standard setup, representing an 85% enhancement (Figure 2a). Notably, this increased robustness consistently maintains the hydrogel in subsequent expansion protocols, where physical stress can be an issue while preventing the jeopardization of cell integrity.

The second and third limitations of TREx for SMLM lie in the homogenization process, where the original workflow utilizes Proteinase K to homogenize the mechanical characteristics of the samples within the hydrogel. Although effective in facilitating isotropic expansion, this method significantly damaged the protein epitopes. Our experiment showed that pre-labeled mitochondria (ATP synthase) and primary cilia (Ac-Tub) exhibited reduced signal intensity and poor signal-to-noise ratios after Proteinase K treatment (Figure 2b; Supplementary Figures S2 and S4a). This phenomenon extends to insufficient fluorescent signals as well as low localization counts in subsequent dSTORM imaging. To address these limitations, we sought to retain epitope accessibility and support post-expansion immunostaining because labeling the sample after hydrogel expansion had been routinely carried out for better signal retention and flexibility for sample handling in other expansion techniques, as demonstrated in ultrastructure expansion microscopy (U-ExM) [24,28].

We thus replaced enzymatic digestion with a denaturation-based homogenization protocol, as shown in the U-ExM strategy, which utilizes ionic detergents under specific temperature and temporal controls (95 °C for 90 min) to denature the tertiary and quaternary protein structures while simultaneously preserving the protein epitopes, which are crucial for antibody recognition. Importantly, this denaturation method enabled robust post-expansion immunolabeling with minimal structural distortion. Comparative imaging of ATP synthase and acetylated tubulin further corroborated that fluorescence signals from denatured, post-labeled samples are of higher intensity and uniformity than those from pre-labeled, proteolyzed samples (Figure 2b,c; Supplementary Figures S2 and S4a). This signal intensity enhancement directly benefits dSTORM performance by providing higher localization counts and increased signal density.

Given the sensitivity of protein denaturation, temporal and temperature control serve as critical parameters that govern whether the hydrogel is fairly homogenized and whether it expands to the desired degree. Systematic screening of a matrix of temperature and incubation time combinations becomes crucial for achieving a true ten-fold expansion at the cellular level (Figure 2d–f). We found that the expansion factor positively correlated with the increasing temperature and treatment time length. Through quantitative analysis of the expansion factor and the amount of structural preservation (Figure 2d–f), we identified 95 °C for 1.5 h as the optimal denaturation condition, which balances mechanical homogeneity and minimizes structural deformation. An additional image result of α-tubulin under the same condition further corroborated epitope preservation, as structures remained intact at 1.5 h but appeared fragmented with 2-h treatment at 95 °C (Supplementary Figure S4b). This condition reliably expands the hydrogel by approximately ten-fold while maintaining intact subcellular morphology and ultrastructural features, with fluorescent signals enhanced via post-immunolabeling, making it suitable for downstream high-resolution imaging.

With these modifications and optimizations, we introduced the plTREx workflow (Figure 1), which maintains the structural integrity of the hydrogel, retains an expansion factor of nearly ten-fold compared to the original TREx workflow, and enhances fluorescent signals with a post-expansion immunolabeling strategy. The aforementioned post-labeling strategy in our protocol is particularly pivotal for downstream SMLM imaging; it promotes higher labeling density, enhances fluorescence, and thus improves blinking statistics in dSTORM.

### 2.2. Ten-Fold Expansion Retention in Less-Polar dSTORM Imaging Conditions

The dSTORM buffer is Tris-based and thiol-rich, significantly less polar than pure water. Exposure to this nonpolar environment induces hydrogel shrinkage since hydrogel expansion relies on interactions with highly polar solvents. Therefore, imaging the pl-TREx-expanded specimen in the dSTORM system requires further action.

**Figure 3:**
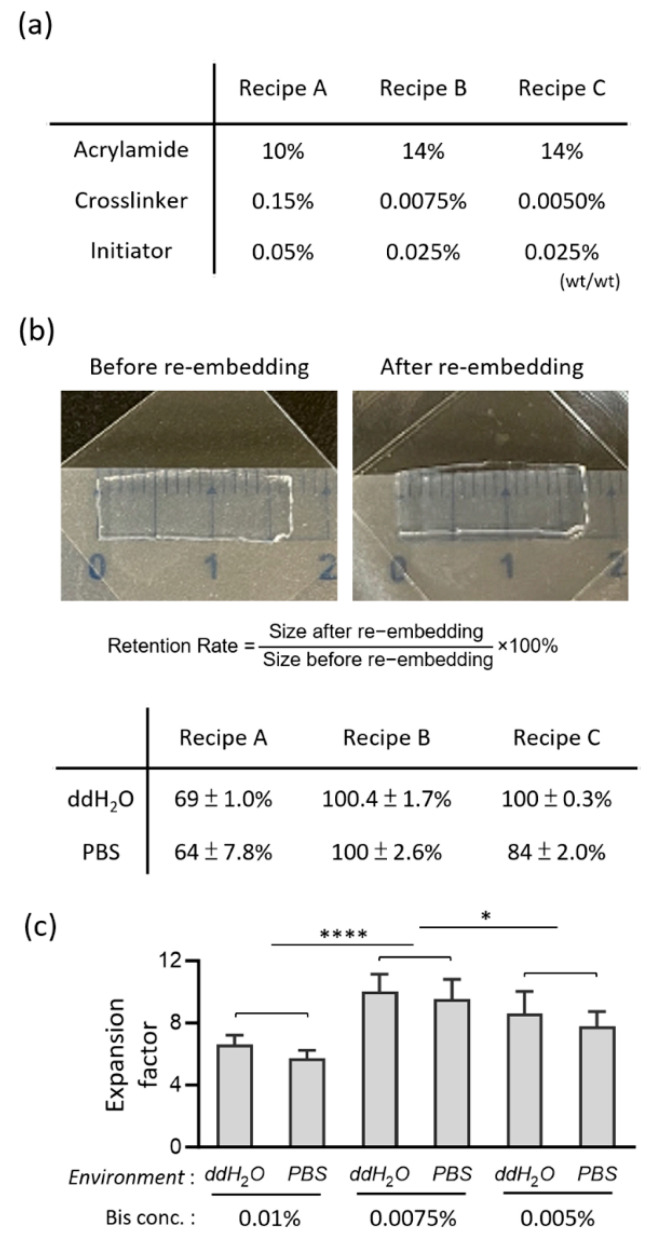
Refined re-embedding strategy for optimal expansion retention of polyacrylamide gels. (**a**) Three recipes for re-embedding neutral gels into the plTREx-expanded samples. (**b**) Expansion retention rate derived from physical measurement of gel dimensions. The hydrogels, after an overnight PBS bath, are placed on coverslips coated with bind-silane and measured with a caliper. Here shows the experiment snapshot of gels post-re-embedding treatment with 0.0075% BIS. Data are presented as mean ± s.d. from 3 independent experiments each. (**c**) Investigation of the expansion factor from the diameter of centrioles. Data are presented as mean ± s.d., based on at least 10 different centrioles. Unpaired two-tailed *t*-test was conducted to assess statistical significance. Significance is indicated as follows: *p* < 0.05 *, *p* < 0.0001 ****.

Hydrogel re-embedding enables the preservation of the structural integrity of the hydrogel up to a certain degree in the dSTORM buffer (Figure 1b). Previous studies found 20–30% shrinkage in the hydrogel after re-embedding [21,29], and our recent study refined the protocol to retain the expansion factor of U-ExM hydrogel in the dSTORM imaging environment up to ~94.5% [22]. Nevertheless, we discovered that directly using the same re-embedding formula (“Recipe A,” Figure 3a) on either TREx or plTREx hydrogel can shrink it by ~30% (“Recipe A,” Figure 3b). Microscopically, using the fluorescently labeled primary cilium as a reference, we found that the scale of expansion was limited to 5× to 6×, and so was the effective spatial resolution, which did not meet our goal of retaining the ten-fold expansion in the dSTORM buffer.

To address this issue, we systematically tuned the re-embedding mixture, altering the concentrations of monomer (acrylamide), crosslinker (N,N′-Methylenebisacrylamide, shortened as BIS), and initiator (Ammonium Persulfate and N,N,N′,N′-Tetramethylethylenediamine) to match the original makeup of monomer solution better, low-crosslinking plTREx hydrogel (Figure 3a; Supplementary Figure S5). Both Recipe B and Recipe C had 14% acrylamide and 0.025% initiator, but they differed in the BIS concentration, where the number in Recipe B (0.0075%) is higher than that in Recipe C (0.005%). Essentially, we formed a hypothesis that matching the BIS concentration in the re-embedding mixture to match the native plTREx matrix would minimize mechanical stress caused by the over-crosslinked network on the sparsely crosslinked hydrogel and thus preserve expansion.

Macroscopically, we measured the hydrogels’ dimensions after re-embedding and discovered that both Recipe B and Recipe C maintained the expansion up to 100% in ddH_2_O, compared to the 69% size retention in Recipe A (Figure 3b). To further distinguish Recipe B and C, we explored the lower ionic interaction of the PBS buffer, in comparison to ddH_2_O, with the hydrogel to select the best candidate to keep the hydrogel’s expansion scale during dSTORM imaging. We observed decreased size retention rates in Recipes A and C. Nonetheless, Recipe B displayed a sharp contrast to the former two recipes, with a 100% retention rate.

Then, to confirm if this expansion retention truly reflects on the microscopic scale, we quantified the expansion factor with fluorescently labeled primary cilia in the plTREx gel across three recipes (Figure 3c). We observed a decrease in expansion from ddH_2_O to PBS buffer across all three conditions and we found ~10-fold expansion in ddH_2_O and ~9.5-fold expansion in the sample re-embedded with the Recipe B formula. Together with macroscopic and microscopic validations and no structural distortion compared to the known EM reference imaging data [30,31,32], these cumulative data suggest that matching monomer concentration, slightly increasing crosslinker concentration, and lowering initiator concentrations in Recipe B compared to the polymerization formula help optimally stabilize the matrix and at the same time not over rigidifying it in dSTORM imaging buffer.

### 2.3. plTREx-dSTORM Enables Nanoscale Imaging of Subcellular Architecture

**Figure 4:**
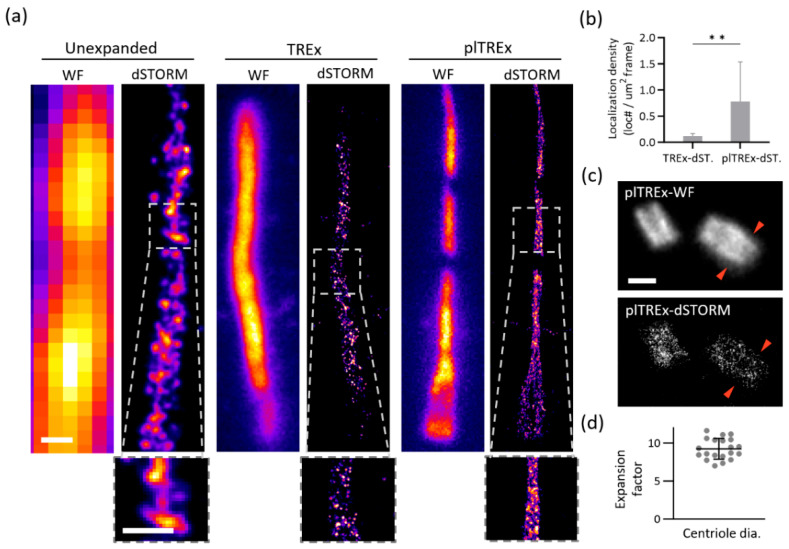
Representative plTREx-dSTORM imaging of primary cilium and centrioles. (**a**) Primary cilium, in cells starved for 48 h, were immunolabeled by acetylated tubulin (AcTub) mouse antibody conjugated to the secondary antibody with CF568. Three imaging methods are shown and labeled each with a pair of widefield and dSTORM imaging of the expanded sample. The box-regions show the magnified features in three respective imaging conditions. (**b**) Quantitative analysis of localization density from TREx-dSTORM and plTREx-dSTORM images. Localization density was defined as the number of localizations per square micrometer per frame. Data are presented as mean ± s.d., based on five super-resolution images for each condition. Unpaired two-tailed *t*-test was conducted to assess statistical significance. Significance is indicated as *p* < 0.01 **. (**c**) Centrioles were immunolabeled by the same antibody pairs as in (**a**) with 20-h starvation to the cell culture. Red arrows show the varying diameter of the structural representations across the two imaging methods. (**d**) pl-TREx expansion factor analysis. Expansion factor is calculated by the measurement of the centriole’s diameter (*N* = 20). Data are presented as mean ± s.d. Scale bar, 200 nm (**a**,**c**).

plTREx-dSTORM delivers exceptional structural clarity in subcellular features, enabling nanoscale delineation of the primary cilium and centriole architectures (Figure 4). Notably, compared with widefield and dSTORM imaging of the control and TREx-expanded samples, plTREx-dSTORM provides a substantial improvement in effective resolution, which, in turn, reflects on the revelation of ultrastructural details of primary cilia (Figure 4a).

In the primary cilium, for example, the connecting region between the basal body and axonemal shaft is difficult to visualize using fluorescence imaging, though it is readily resolved by electron microscopy [30,31,32]. In widefield imaging under the unexpanded condition, the primary cilium appears blurry and poorly defined. Although dSTORM imaging improves structural representation, the spatial resolution and labeling density remain insufficient to reveal the distinction. TREx-expanded cilium in widefield shows a slightly more defined structure than the control. Further dSTORM imaging enables resolution improvement yet fails to visualize the minute patterns. Notably, the plTREx-dSTORM result significantly increases localization counts by revealing denser puncta throughout the cilium, providing a detailed exhibition of axonemal architecture (Figure 4b). This is especially evident in the inset, where dense and continuous signal tracks are only visualized in plTREx-dSTORM, in stark contrast to the fragmented signals in dSTORM of unexpanded and TREx-expanded samples.

Similarly, plTREx-dSTORM reveals the architectural details of centrioles, furthering the investigative effort of plTREx (Figure 4c). In contrast, plTREx-dSTORM of centrioles shows more clearly defined structural contours. Specifically, the red arrows shown across the two figures further suggest that plTREx-dSTORM can resolve sharper contours than the widefield of plTREx, where the outline of the structure is blurred and the edge is blunt. To verify the true expansion achieved in plTREx-dSTORM, the diameter of centrioles in the RPE-1 wildtype cell line was quantitatively analyzed across 20 samples to calculate the expansion factor achieved in microscopic measurements (Figure 4d). The centriolar diameter measurements in the plTREx-dSTORM result indicate an expansion factor of 9.3, closely matching the macroscopic expansion size of the gels. To further verify expansion stability over the course of imaging, we tracked the expansion factor between the first and last primary cilium imaged during a ~2-h dSTORM session. The values remained consistent (~9.5× to ~9.3×), a 97% expansion retention rate and no evidence of shrinkage or distortion under prolonged exposure to the photoswitching buffer. Together, these analytical and imaging data corroborate that our Ex-dSTORM preparation strategy facilitates the promotion of structural fidelity in the polyacrylamide hydrogel. Moreover, the aforementioned plTREx-dSTORM imaging data of primary cilium and centrioles reflects the impressive degree of structural precision directly associated with the expansion factor achieved.

Given the retained ten-fold scale and the resolution capacity of dSTORM, plTREx-dSTORM achieves a theoretical effective resolution of ~2.6 nm and localization precision of ~1 nm (Supplementary Figure S6). The effective resolution is obtained by dividing the 24.3 nm resolution, determined through FRC analysis, by the 9.3-fold expansion. Similarly, the effective localization precision of our system is calculated as ~9 nm divided by 9.3. This validates the optics-based imaging resolution, which approaches that of EM and enables investigation of nanometer-scale features such as protein ultrastructure. Through our expansion strategy with a refined re-embedding protocol, we successfully implemented the sample, which expanded nearly ten-fold, in the dSTORM imaging system—a favorable tool for biological imaging that localizes individual protein molecules with precision, thereby reassuring and strengthening optical imaging for true and accurate visualization.

## 3. Discussion

We have systematically developed plTREx and promoted its combination with dSTORM without sacrificing the expansion effort. Our plTREx workflow enables improved mechanical stability to address fragility, provides fluorescent signal display through a post-labeling strategy, and achieves specimen expansion nearly ten-fold. With dSTORM, we further optimized the re-embedding strategy to achieve plTREx expansion capability across ddH_2_O and the Tris-based, thiol-rich imaging buffer. While previous studies have combined ExM with dSTORM, they typically achieved expansion factors of around 4×. Moreover, they faced challenges such as gel shrinkage in photoswitching buffers, even after re-embedding [19,33]. Our approach addresses these limitations by enabling a ten-fold expansion compatible with dSTORM imaging. This represents an exciting advance over previous Ex-SMLM approaches, particularly those limited to four-fold expansion strategies with dSTORM, which has reduced effective resolution due to insufficient expansion. The plTREx-dSTORM opens a new door for molecular-level investigations, like protein organizations, as evident in the high-resolution visualization of the ultrastructural context in centriolar features. This suggests the potential of this superresolution solution to investigate an array of biomolecules that other optical modalities may not efficiently execute, leveraging a deeper understanding of biology down to the molecular scale.

Compared to other expansion superresolution methods, plTREx-dSTORM offers a number of advantages in its imaging performance (Supplementary Table S1). For starters, Ex-STED generally achieves lower effective resolution than Ex-SMLM, where plTREx-dSTORM in the study belongs to, due to its different mechanism to bypass the diffraction limit of light. Ex-dSTORM, particularly the combination of dSTORM with ExM or U-ExM, offers about 4× expansion—with optimal expansion retention taken into account—which inherently limits the effective resolution to breakthrough below the ~5–10 nm range. plTREx-dSTORM, on the other hand, achieves a higher expansion factor close to 10× with enhanced labeling density (insets in Figure 4a) and thus an enhanced effective localization precision to ~1 nm. In addition, it is important to note that while direct signal-to-noise ratio (SNR) comparisons are not feasible across different expansion scales, SNR in U-ExM samples may appear higher due to increased labeling density, assuming the total amount of fluorophores remains constant. Moreover, we carefully tuned the denaturation conditions to mitigate the potential over-expansion artifacts: structures imaged under 95 °C for 1.5 h retain structural continuity while 2 h treatment introduce detectable breakages of the ciliary axoneme (Figure 2e).

The performance of plTREx-dSTORM is highly dependent on fine-tuning the polyacrylamide network, especially the temperature and time control in denaturation and re-embedding formulation. To begin with the gel handling, instead of performing mechanical testing such as Young’s modulus measurement, we adopted a deformation analysis approach, as described in the previous study (Damstra et al.), serving as a reproducible proxy to quantify improvements in gel physical stability and handling (Figure 2a). Secondly, the slight imbalances in each variable might lead to gel shrinkage, deviating from our goal of expansion retention. Denaturation-based homogenization, while preserving epitope accessibility to enable post-labeling, poses a risk of structural distortion when temperature and duration exceed the optimal threshold. Thirdly, it is difficult to image the target proteins on the same focal plane because the cells and molecules inside the expansion matrix are expanded three-dimensionally. In addition, there are practical concerns about antibody penetration in dense specimens or antibody labeling efficiency. Together, these aspects underscore the method’s technical sensitivity and the extent of its current applications. We further demonstrated the versatility of the plTREx-dSTORM workflow to image additional markers, including the Golgi apparatus (GM130) and cytoskeletal protein α-Tubulin (Supplementary Figure S7). We introduced a powerful expansion strategy that can extend to other superresolution modalities. These include stimulated emission-depletion microscope (STED) and SMLM (such as PALM and DNA-PAINT). Or one can use plTREx to visualize protein molecules, cell samples, or tissue slices with the advantages that expansion microscopy could offer—a convenient, cost-effective, and robust approach for nanoscale structural imaging using a widefield or confocal microscope.

In practice, we observed a clear distinction in the basal body and axoneme in each primary cilium—the extension of the ciliary architecture from the proximal end of the mother centriole (Figure 4). As plTREx-dSTORM achieves a clear resolution of the basal body-axoneme transition, a feature typically resolved only by electron microscopy. This method enhances optical imaging for visualizing organelles and probing sub-organelle and ultrastructural details. For example, with dual gain in expansion and localization precision of plTREx-dSTORM imaging, we can observe the ciliary growth trend, deciphering architectural nuances like the two and five trendlines on the same primary cilium but in different imaging methods, plTREx and Ex-dSTORM (Supplementary Figure S8). Going far beyond the nomenclature of the new imaging techniques, we have developed plTREx-dSTORM, which contributes to enhanced imaging capability within the Ex-SMLM framework. Importantly, plTREx-dSTORM pushes the boundary of superresolution imaging and bridges the gap between optical imaging and electron microscopy, leading to potential molecular exploration in biological and medical research.

## 4. Materials and Methods

### 4.1. Reagents

Bovine serum albumin (BSA, A9647, Sigma-Aldrich, St. Louis, MO, USA), Tween 20 (P137, Sigma-Aldrich), Methyl alcohol (methanol, 15306121, Macron, Center Valley, PA, USA), Phosphate buffered saline (10X PBS, 70011044, Gibco, Grand Island, NY, USA), Dimethyl sulfoxide (DMSO, D8418, Sigma-Aldrich), Sodium acrylate (SA, 97%, 408220, Sigma-Aldrich), Acrylamide (AA, 40%, A4058, Sigma-Aldrich), Acrylamide/Bis-acrylamide (30%, 29:1, 1610156, Bio-Rad, Hercules, CA, USA), N,N,N′,N′-Tetramethylethylenediamine (TEMED, 1610801, Bio-Rad), Ammonium persulfate (APS, 1610700, Bio-Rad), Sodium dodecyl sulfate (SDS, 0227, VWR Life science, Radnor, PA, USA), Sodium chloride (NaCl, 31434, Sigma-Aldrich), Tris (1.5 M, pH 8.8, J831, VWR Life Science), Bind-silane (abx082155, Abbexa, Cambridge, UK), Acetic acid (33209, Sigma-Aldrich), and Ethanol (absolute, ≥99.8%, 32221, Sigma-Aldrich).

### 4.2. Cell Culture

Human retinal pigment epithelial cells (hTERT RPE-1, shortened as RPE-1, ATCC-CRL-4000, Manassas, VA, USA) were cultured at 37 °C with 5% CO_2_ in the environment. The culture medium is comprised of Dulbecco’s modified Eagle’s medium (DMEM)/F-12 mixture medium with L-glutamine, HEPES (1:1; 11330-032, Gibco, Thermo Fisher Scientific, Grand Island, NY, USA), 10% fetal bovine serum (FBS, SH3010903, Hyclone, Cytiva, Logan, UT, USA), sodium bicarbonate (NaHCO_3_, S6014, Sigma-Aldrich), and 1% penicillin-streptomycin (15140122, Gibco, Thermo Fisher Scientific).

### 4.3. Sample Preparation for TREx

RPE-1 cells were transferred and cultured on coverslips coated with poly-L-lysine before fixation, and 24–48 h serum starvation was applied to induce cilium formation. Fixation was performed with cold methanol at −20 °C for 10 min.

Then, antibody staining was performed to label the structure of interest specifically in fluorescence microscopy. Primary antibodies used in this study were acetylated tubulin (rabbit IgG; Catalog No. 5335S; Cell Signaling, Danvers, MA, USA) for primary cilia and centrioles visualization, and ATP synthase (mouse IgG; Catalog No. ab109867; Abcam, Cambridge, UK), for in situ drift correction in the later section titled “Drift Correction.” Secondary antibodies applied were CF568 (dilution 1/100, Goat Anti-Rabbit IgG(H+L); Catalog No. 20098; Biotium, Fremont, CA, USA) and Alexa Fluor 488 (dilution 1/100, Donkey anti-Mouse IgG (H+L); Catalog No. A21202; Invitrogen, Waltham, MA, USA).

After antibody staining is performed, gelation (or polymerization) was carried out via incubating each coverslip at 37 °C with a monomer solution (14% (*w*/*w*) SA, 10% (*w*/*w*) AA, 0.005% (*w*/*w*) BIS in PBS) supplemented with 0.25% TEMED and 0.25% APS. After pre-polymerization on the ice for 1 min, the chamber was transferred into the incubator for gelation at 37 °C for 1 h. After gelation, coverslips with hydrogel were placed in a fresh digestion buffer (proteinase K diluted 1/100 in digestion buffer) for 3 h (TREx) at RT. Detached hydrogels were washed twice in fresh PBS at room temperature for 15 min each. Finally, the polymer gel matrix is expanded in fresh, double-deionized water (ddH_2_O) at least three times, each for 30 min, with gentle shaking, and then incubated overnight in ddH_2_O.

### 4.4. Sample Preparation for plTREx

Instead of Proteinase K digestion and antibody staining before gelation, the cells on coverslips were directly incubated at 37 °C with the same TREx monomer solution (described in the last section) and, then, the polymerized gel matrix with cells embedded was bathed in denaturation buffer (200 mM SDS, 200 mM NaCl in 50 mM Tris (pH 8.8) for 15 min at RT with gentle shaking to detach the hydrogels from the coverslips. Then, the pl-TREx hydrogels were put in fresh denaturation buffer and treated at 95 °C for 1–2 h (note that this is the optimal condition from testing denaturation conditions at 85–95 °C for 1.5–2 h). Afterwards, the hydrogels were transferred to a Petri dish and were expanded in fresh, double-deionized water at least three times with gentle shaking until an expansion plateau was reached.

Subsequently, the hydrogels were kept in PBS for immunostaining and then trimmed to approximately 1 cm × 0.5 cm dimensions (width × length) before being placed into Eppendorf tubes. Primary antibodies (acetylated tubulin and ATP synthase, same products as used in TREx) were diluted 1:200 in PBS supplemented with 2% BSA, and 100 µL of the antibody solution was added to each trimmed gel. Samples were incubated at 37 °C for 3 h on an orbital shaker (about 80 rpm). Then, samples were washed three times with 0.1% Tween-20 in PBS (PBST) for 10 min each with gentle shaking. Secondary antibody staining was performed following this with 100 µL of a 1:200 dilution of secondary antibodies (CF568 and Alexa Fluor 488, same products as used in TREx) in the mixture of 2% BSA in PBS. Similar to the previous operation, the samples were washed three times with 0.1% PBST for 10 min. Finally, the samples were transferred to 15 cm dishes and bathed in fresh deionized water, which was exchanged at least three times until the expansion plateau was reached. For widefield imaging of the expanded samples, the hydrogel was trimmed to approximately 2 cm × 2 cm dimensions (width × length) and placed in water to prevent shrinkage during imaging.

### 4.5. Hydrogel Re-Embedding

The immunolabeled, expanded pl-TREx hydrogels were incubated in fresh re-embedding solution (14% (*w*/*w*) AA, 0.0075% (*w*/*w*) BIS, 0.025% (*w*/*w*) TEMED, 0.025% (*w*/*w*) APS in ddH_2_O) for 25 min and were exchanged again with another fresh re-embedding solution, both with gentle shaking. In parallel, a fresh bind-silane coating solution (5 μL bind-silane, 8 mL absolute ethanol, 200 μL acetic acid, and 1.8 mL ddH_2_O) was prepared. Few coverslips were prepared and washed with ddH_2_O and absolute ethanol, followed by rinsing with bind-silane coating solution. After the coating solution had completely evaporated, the coverslips were washed with absolute ethanol and then left to air-dry. The hydrogel samples, after the re-embedding bath, were transferred onto these coverslips and placed together in a nitrogen-filled, humidified chamber at 37 °C for 1.5 h incubation. The result was the expanded pl-TREx hydrogel being crosslinked with another neutral acrylamide gel for stabilization in the dSTORM imaging buffer. The re-embedded gel was washed three times with ddH_2_O for 20 min each. Note that the three re-embedding solutions were tested, except for the optimal one to retain expansion factor well close to 10 described already, 10% (*w*/*w*) AA, 0.05% (*w*/*w*) BIS, 0.5% (*w*/*w*) TEMED, 0.5% (*w*/*w*) APS in ddH_2_O and 14% (*w*/*w*) AA, 0.005% (*w*/*w*) BIS, 0.025% (*w*/*w*) TEMED, and 0.025% (*w*/*w*) APS in ddH_2_O.

### 4.6. plTREx-dSTORM Imaging and Validation

The dSTORM image acquisition was conducted using a custom-built setup based on a commercial inverted microscope (Eclipse Ti-E, Nikon, Tokyo, Japan) that was equipped with a laser merge module (ILE, Spectral Applied Research, Richmond Hill, ON, Canada) and a focus stabilizing system. The setup included four light sources that were individually controlled, i.e., a 561 nm laser (Jive 561 150 mW, Cobolt, Solna, Sweden), a 488 nm laser (OPSL 488 LX 150 mW, Coherent, Santa Clara, CA, USA), and a 405 nm laser (OBIS 405 LX 100 mW, Coherent), which were homogenized using a Borealis Conditioning Unit (Spectral Applied Research, Richmond Hill, ON, Canada) and focused onto the back focal plane of an oil-immersing objective (100X 1.49, CFI Apo TIRF, Nikon) for widefield illumination of the samples. During the dSTORM image acquisition, the 561 nm laser lines was operated at a high intensity of approximately 3 kW/cm^2^ to quench most of the fluorescence from CF568. To convert a portion of fluorophores from a dark to a fluorescent state, a weak 405 nm laser beam was introduced. The 488 nm laser line was intermittently switched on every 800 frames for in situ drift correction. Fluorescent signals were detected using a quad-band filter (ZET405/488/561/640 mv2, Chroma, Bellows Falls, VT, USA) and recorded on an electron-multiplying charge-coupled device (EMCCD) camera (Evolve 512 Delta, Photometrics, Tucson, AZ, USA) with a pixel size of 93 nm. For single-color imaging, the CF568 channel was acquired using a combination of quad-band and short-pass filters (BSP01-633R-25, Semrock, Rochester, NY, USA). Typically, 15,000–30,000 frames were acquired at a rate of 50 fps for each dSTORM image. The individual single-molecule peak position was then localized using the MetaMorph Superresolution Module (Molecular Devices, San Jose, CA, USA) based on a wavelet segmentation algorithm. The superresolution images were cleaned using a Gaussian filter of 0.7–1 pixel. Prior to imaging, the immobilized re-embedding hydrogels were immersed in an imaging buffer (Tris-HCl, NaCl (TN) buffer at pH 8.0, 10–100 mM mercaptoethylamine (MEA) at pH 8.0, and an oxygen-scavenging vsystem consisting of 10% glucose (G5767, Sigma-Aldrich), 0.5 mg mL^−1^ glucose oxidase, and 40 μg mL^−1^ catalase). To validate the system resolution, Fourier Ring Correlation (FRC) analysis was conducted on plTREx-dSTORM images of Ac-Tubulin–stained centrioles and cilia using the GDSC SMLM plugin, an ImageJ (1.53q)-based software tool [33]. The resolution was estimated by applying a threshold value of 1/7 on the FRC curve to identify the cutoff spatial frequency, thereby determining the corresponding spatial resolution. Additionally, localization precision was quantified with the same plugin through detection and fitting of individual single-molecule bursts.

### 4.7. Drift Correction and Post-Imaging Processing

The dSTORM imaging often requires post-correction of acquired images because the localized puncta are subjected to drift after tens of thousands of frames acquired over several minutes. The in-situ drift correction was introduced. ATP Synthase (mouse IgG; Catalog No. ab109867; Abcam) was used as a marker protein, which was intermittently excited and emitted signals during acquisition of the target channel of CF568, and signals from the marker channel were corrected by an ImageJ plugin to correlate the pattern shifted over the acquired data. Once the data were corrected, a homemade code, based on LabVIEW (National Instruments, Austin, TX, USA), MATLAB (MathWorks, Natick, MA, USA), and ImageJ (NIH, Bethesda, MD, USA), was used to eliminate the marker signals.

### 4.8. Data Analyses and Reproducibility

This study mainly included deformation analysis, macroscopic expansion factor analysis, and microscopic expansion factor analysis. For starters, deformation analysis was carried by measuring three different thicknesses, determined by the number of coverslips stacked in the gelation chamber of the expanded hydrogels (Supplementary Figure S3). Expanded hydrogels were cut in half to achieve a semicircular shape and put on the board to measure the vertical deviation Δ*r* from the base to the arc relative to the gel radius *r* (Figure 2a). Secondly, the macroscopic expansion factor analysis was determined by dividing the measurements of the gel dimensions before and after the re-embedding treatment (Figure 3b). Lastly, the microscopic expansion factor was determined by dividing the diameter measured from the centrioles in expanded and unexpanded samples (Figure 2d, Figure 3c and Figure 4d). In addition, all widefield, dSTORM, TREx-dSTORM, and plTREx-dSTORM images panels were repeated at least three times, and representative images were shown in this paper (Figure 2b,e,f, and Figure 4a,c; Supplementary Figures S1, S2, S4 and S6–S8).

## Data Availability

All the data supporting the findings described in this study are available within the article and supplementary information from the corresponding author upon reasonable request.

## Supplementary Materials

The following supporting information can be downloaded at: https://www.mdpi.com/article/10.3390/gels11070491/s1, Table S1: Summary table comparing plTREx-dSTORM to other Ex-SMLM methods, U-ExM-dSTORM (Chang et al., 2023 [22]) and Ex-SMLM (Zwettler et al., 2020 [21]), and Ex-STED (Gao et al., 2018 [23]); Figure S1: Issues identified in the original ten-fold ExM workflow; Figure S2: Comparison between pre-labeled versus post-labeled ATP synthases in TREx (sample treated with proteinase K digestion) and plTREx (sample treated with heat-induced denaturation) protocols; Figure S3: Schematic diagram of the gelation environment; Figure S4: Preservation of plTREx-expanded structures with different homogenization methods; Figure S5: Simplified process flow from hydrogel formation to re-embedding treatment; Figure S6: Validation of resolution; Figure S7: Demonstrations of post-expansion immunolabeling versatility in plTREx protocol; Figure S8: Growth trend fitting of primary cilia in TREx and TREx-dSTORM.
