# Supplementary material for: Pushing Optical Resolution to the Few-Nanometer Scale via dSTORM Imaging of Expanded Specimen–Gel Composites
Source: Gels. 2025 Jun 25;11(7):491. doi: 10.3390/gels11070491 (PMC12294864; doi:10.3390/gels11070491)
Supplement: Supplementary file 1 [file gels-11-00491-s001.zip › gels-3703534-supplementary.pdf]

Supporting Information

Pushing Optical Resolution to the Few-Nanometer Scale via dSTORM Imaging of Expanded Specimen–Gel Composites

Jimmy Ching-Cheng Hsu[1,2], T. Tony Yang*[1,2]

[1]Department of Electrical Engineering, National Taiwan University, Taipei, 10617, Taiwan

[2]Graduate Institute of Biomedical Electronics and Bioinformatics, National Taiwan University, Taipei, 10617, Taiwan

*Corresponding author:

T. Tony Yang

tonyyang@ntu.edu.tw

Supplementary Table 1. Summary table comparing plTREx-dSTORM to other Ex-SMLM methods, U-ExM-dSTORM (Chang et al., 2023) and Ex-SMLM (Zwettler et al., 2020), and Ex-STED (Gao et al., 2018). Refer to Figure 3a for Recipe A and Recipe B re-embedding formulations.

Method	Expansion Factor	Effective Resolution (nm)	Expansion Retention in dSTORM Buffer
plTREx-dSTORM (this study)	~9.3×	~2 nm	~100% (Recipe B)
TREx-dSTORM	~5.0×	~4 nm	~69% (Recipe A)
U-ExM-dSTORM (Chang et al.)	~4×	~5 nm	~94.5% (Recipe A)
Ex-SMLM (Zwettler et al., 2020)	~3.2x	~6 nm	~70-80% (Recipe A)
Ex-STED (Gao et al., 2018)	~4x	~10 nm	Not applicable

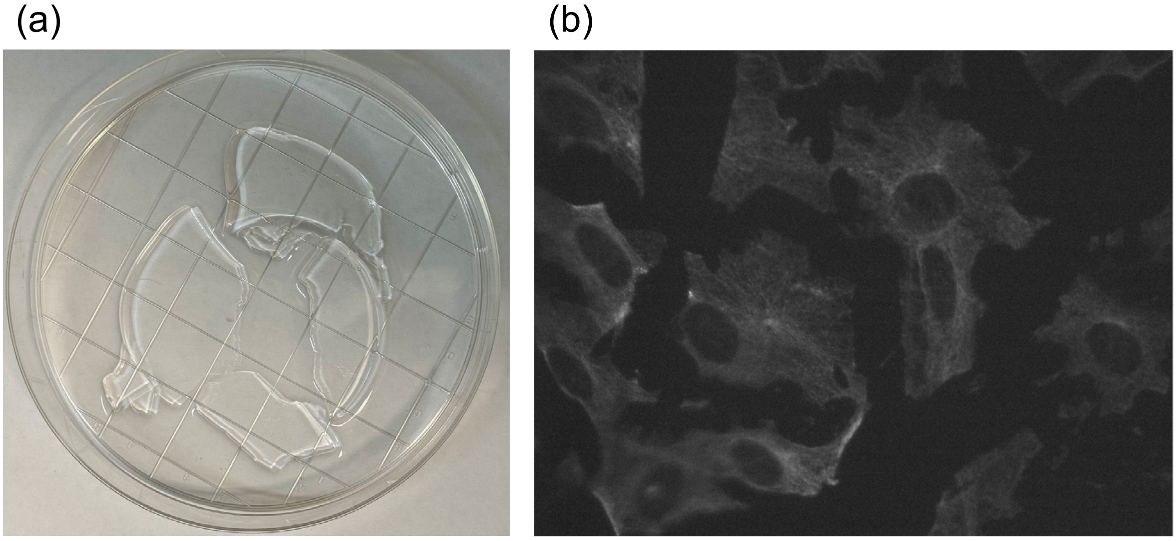

Supplementary Figure 1. Issues identified in the original ten-fold ExM workflow. (a) Expanded hydrogel breakage due to weak mechanical properties. The image shows the 15 cm-diameter dish. (b) Compromised cell integrity is displayed in widefield imaging of the cytoskeleton.

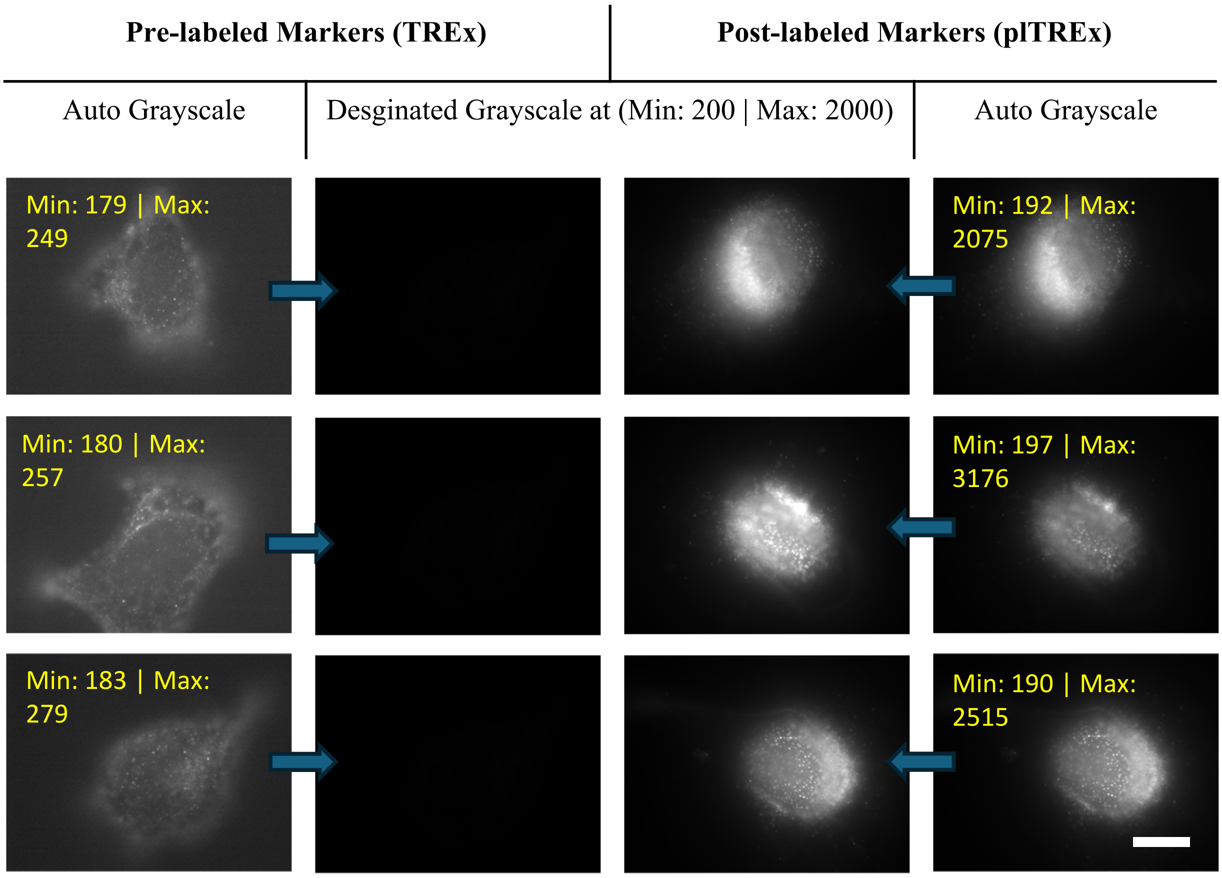

Supplementary Figure 2. Comparison between pre-labeled versus post-labeled ATP synthases in TREx (sample treated with proteinase K digestion) and plTREx (sample treated with heat-induced denaturation) protocols. Scale bar, 10 μm.

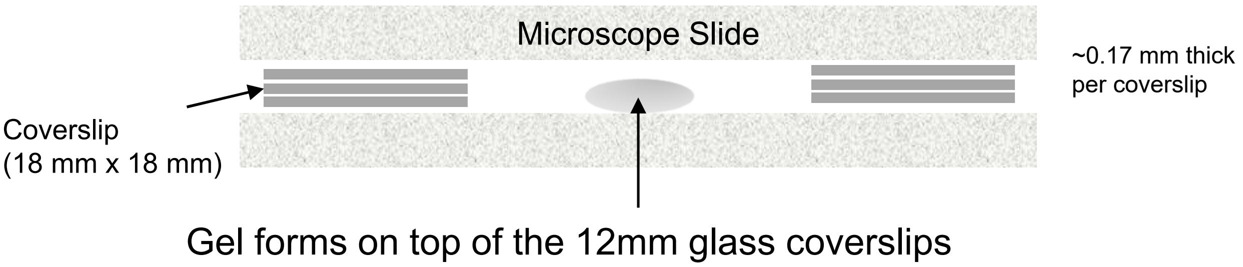

Supplementary Figure 3. Schematic diagram of the gelation environment. Two microscope slides sandwich a volume of polymerization mixture (about 28-60 μL) with customizable height, supported by the number of coverslips stacked in each pillar.

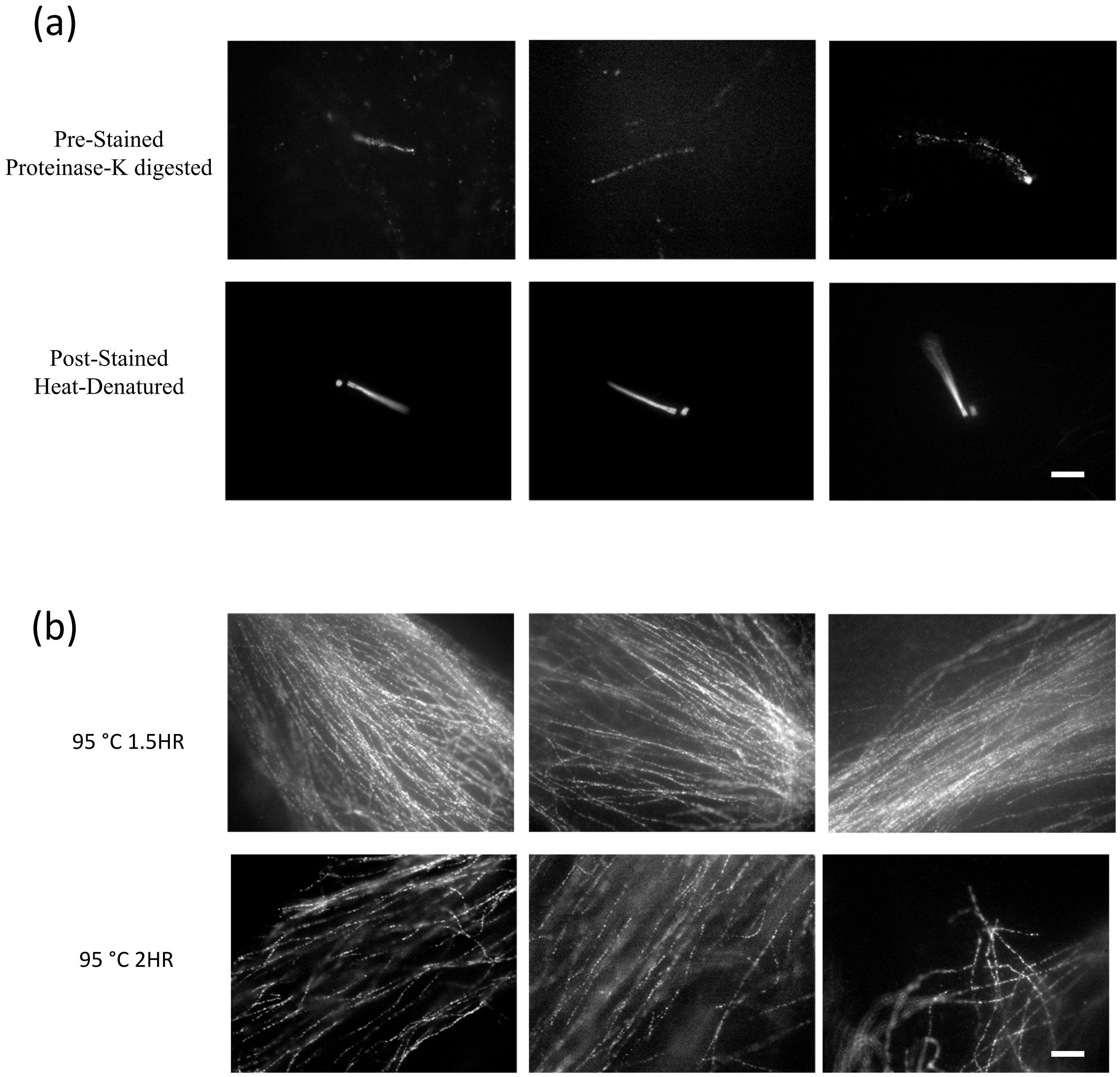

Supplementary Figure 4. Preservation of plTREx-expanded structures with different homogenization methods. (a) Comparison between pre-labeled, proteinase-k digested versus post-labeled, heat-denatured primary cilia in TREx and plTREx protocols. (b) Comparison between alpha-tubulin denatured in plTREx protocols with 95 °C denaturation for 1.5 and 2h. Scale bar, 10 µm (a,b).

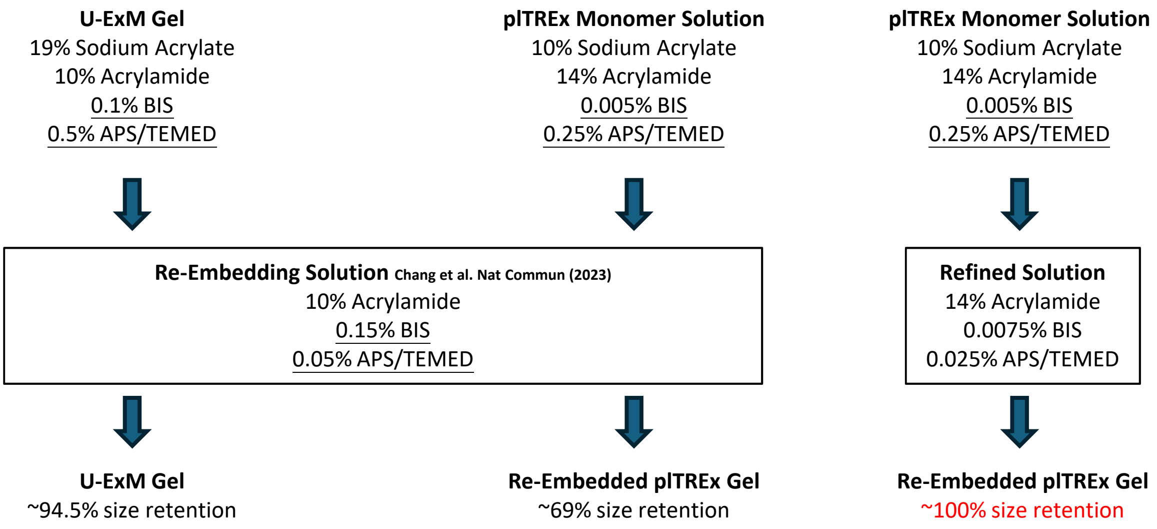

Supplementary Figure 5. Simplified process flow from hydrogel formation to re-embedding treatment. Each column (three columns in total) is followed by the order of treatments with detailed chemicals–Monomer solution formula, re-embedding formula, and the final expansion retention rate.

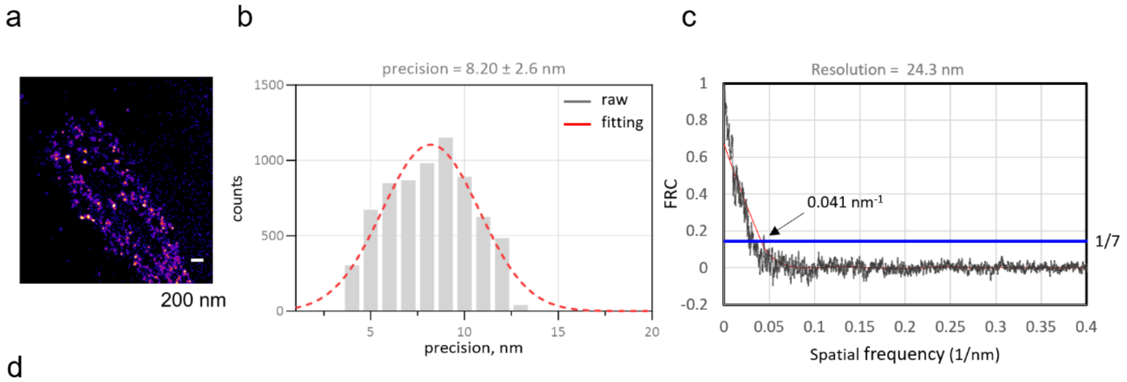

d

	Precision (nm)	Resolution (nm)
Original scale	8.20 ± 2.6	24.3
Biological scale 1/9.3*	0.88 ± 0.28	2.61 (shortest distance between two labeled molecules that can be distinguished with Ex-dSTORM)

* 9.3 is the averaged expansion factor in this work.

Supplementary Figure 6. Validation of resolution. (a) Representative Ex-dSTORM image of Ac-Tub in RPE-1 cell for the resolution analysis. (b) Histograms analysis of localization precision from single molecules per switching event. (c) FRC analysis on the image shown in (a) with a threshold designated to 1/7, as indicated in the blue line. (d) Table of resolution data from (b) and (c) along with an additional row of effective resolution value incorporated with the expansion factor gained in this study.

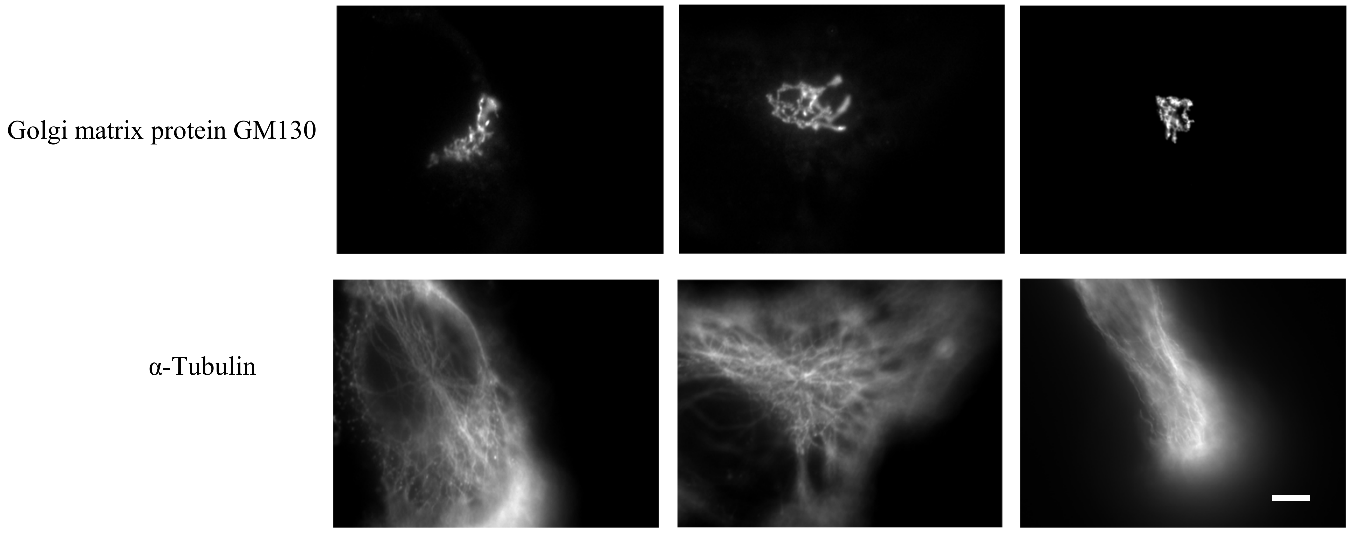

Supplementary Figure 7. Demonstrations of post-expansion Immunolabeling versatility in pITREx protocol. Here shows Golgi apparatus characterized with GM130-AF488 antibody conjugate and cytoskeleton structure characterized with AlphaTub-CF568. Scale bar, 10 μm.

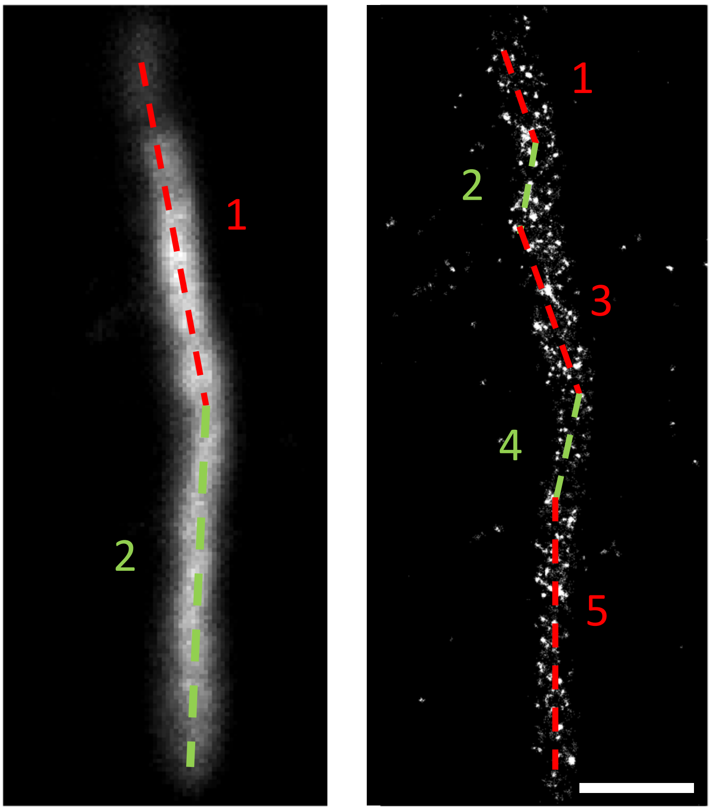

Supplementary Figure 8. Growth trend fitting of primary cilia in TREx and TREx-dSTORM. The trend of the growing direction of the acetylated tubulin was fitted with lines. Red and green dotted lines, along with their corresponding numberings. Scale bar, 500 nm.
